# NRS2002 score as a prognostic factor in solid tumors treated with immune checkpoint inhibitor therapy: a real-world evidence analysis

**DOI:** 10.1080/15384047.2024.2358551

**Published:** 2024-05-30

**Authors:** Wanfen Tang, Chenghui Li, Dong Huang, Shishi Zhou, Hongjuan Zheng, Qinghua Wang, Xia Zhang, Jianfei Fu

**Affiliations:** a Department of Medical Oncology, Jinhua Municipal Central Hospital, Hangzhou, China; b Department of Colorectal Surgery, Jinhua Municipal Central Hospital, Hangzhou, China

**Keywords:** Tumor immune checkpoint inhibitor therapy, cancer nutrition, Nutritional Risk Screening (NRS) 2002, real-world research

## Abstract

To observe the antitumour efficacy of programmed death 1 (PD-1) inhibitors in the real world and explore the relationship between NRS2002 score or other clinical characteristics and immunotherapy efficacy, we retrospectively analyzed 341 tumor patients who received immune checkpoint inhibitor (ICI) treatment at one center. A total of 341 solid tumor patients treated with ICIs from June 2018 to December 2021 were retrospectively included in this study. Patient characteristics, ICI responses, and survival status were documented, and the relationships between clinical factors and survival were analyzed. Among all patients, the median progression-free survival (PFS) was 5.8 months, and the median overall survival (OS) was 12.5 months. The Performance Status (PS), NRS2002 score, The Naples Prognostic Score (NPS), Lymphocyte and C-reactive protein ratio (LCR), line of therapy, and nutritional support were significantly related to PFS or OS according to univariate analysis. The median PFS and OS were significantly better in the group without nutritional risk (NRS2002 0–2) than those with nutritional risk (NRS2002 ≥ 3) (PFS: HR = 1.82, 95% CI 1.30–2.54, *p* value < .001; OS: HR = 2.49, 95% CI 1.73–3.59, *p* value < .001). Cox regression analysis revealed that the NRS2002 score was an independent prognostic factor for both PFS and OS. The objective response rate (ORR) in the group at nutritional risk was lower than that in the group without nutritional risk (8.33% and 19.71%, respectively, *p* value = .037). Patients at nutritional risk according to the NRS2002 score at initial treatment had a poorer prognosis than those without nutritional risk. The NRS2002 could be used as a preliminary index to predict the efficacy of immune checkpoint inhibitor therapy.

## Introduction

Immunotherapy for tumors refers to the use of one’s own immune cells to suppress and kill tumor cells, which has become one of the important research directions in tumor therapy. Among them, blocking immunecheckpoint pathway therapy has become a hot research topic.^1^ PD-L1 is expressed by antigen-presenting cells, including human peripheral blood monocytes, and activated human and murine dendritic cells.^2^ PD-1 mediates immune suppression and is a key immunecheckpoint receptor expressed by activated T cells.^3^ Its ligand, PD-L1, is an anti-inflammatory cytokine responsible for T cell activation, proliferation, and cytotoxic secretion in cancer to produce anti-tumor immune responses.^4^ Immune checkpoint inhibitors promote tumor cell death by blocking immune checkpoint pathways, reactivating T-cell-mediated antitumor immunity, and reversing immune escape.^5^ PD-1 inhibitors in China include pembrolizumab, nivolumab, atezolizumab, durvalumab, toripalimab, camrelizumab, sintilimab, tislelizumab and envafolimab. These drugs have proven effective in treating various malignant tumors, including lung cancer, malignant melanoma, gastric cancer, and esophageal carcinoma.^6–9^ In addition, there are still hundreds of clinical trials on treating different malignancies with ICIs worldwide, creating new options for treatment strategies of malignancies. Although ICIs have shown efficacy and value in many registered clinical studies, patient situations are often more complicated in the real world. The question of whether ICIs can have the same effect as observed in registered clinical studies must be answered with more real clinical case data for verification; it is worth exploring what clinical characteristics of patients benefit more from immunotherapy. Based on this goal, we retrospectively analyzed real clinical cases in which patients received ICI treatment at our center to determine the efficacy and benefits of real-world ICI treatment.

## Materials and methods

### Patient

In this study, we retrospectively included 341 patients with solid tumors who underwent PD-1 inhibitor immunotherapy at the Department of Oncology, Affiliated Jinhua Hospital, Zhejiang University School of Medicine, between June 2018 and December 2021. Pathology confirmed that all patients had tumors.

Each patient agreed to receive ICI; however, as this was a retrospective study, the study did not include the patient’s informed consent signature. This study complied with the ethical standards of the Institutional Research Council and the Declaration of Helsinki. This study was approved by the Institutional Review Committee of JinYi Group Medical Center.

### Data collection

The data of all patients were collected through the Haitai electronic medical record system of the hospital, the image data of patients were collected through the PACS, and the data in the medical record system were automatically captured through standardized software, including general information (sex, age, height, weight, Eastern Cooperative Oncology Group Performance Status (ECOG PS), Nutritional Risk Screening 2002 (NRS2002), disease-related information (serum albumin, total cholesterol level, neutrophil count, lymphocyte count, monocyte count, platelet count, c-reactive protein, comorbidities, primary tumor site and metastatic site), treatment information (treatment time, treatment regimen, number of lines of therapy and nutritional support or not), and efficacy (investigator-assessed efficacy, time to disease progression, and overall survival time). NPS was calculated using the Galizia team’s method based on four values: serum albumin (ALB), total cholesterol level (TC), neutrophil – lymphocyte ratio (NLR), and lymphocyte – monocyte ratio (LMR). The scores were divided into two groups, with 1 or 2 points as the first group and 3 or 4 points as the second group.^10^ The systemic immune inflammatory index (SII) was calculated as the platelet count×neutrophil count/lymphocyte count. LCR=lymphocyte count/c-reactive protein.

In this retrospective study, the efficacy of immunotherapy was evaluated by investigators according to the Response Evaluation Criteria in Solid Tumors (RECIST, version 1.1).^11^ The objective response rate (ORR) was defined as the proportion of patients who achieved complete or partial remission after receiving ICI treatment. PFS was defined as the period from the first ICI treatment to the initiation of disease progression or to death. Overall survival (OS) was defined as the period from the first ICI treatment to the date of the last follow-up appointment or death.

### Statistical analysis

All the statistical analyses were performed using RStudio version 4.3.2 (http://www.r-project.org/). Time-to-event analysis was plotted on Kaplan – Meier curves, and a log-rank test was carried out to evaluate the survival differences between groups. Variables with a *p* value of less than .05 in the univariate analyses were included in multivariate Cox regression with a stepwise method to determine predictor factors for OS or PFS. In all analyses, a *p* value of < .05 was considered indicative of a significant difference.

## Results

### Patient characteristics

A total of 341 patients with solid tumors who had undergone at least one dose of ICI with or without chemotherapy were enrolled in this study; the median age of was 65 years (range from 22 to 91 years), and 253 patients (74.19%) were males while 88 (25.81%) were females. A total of 248 patients received first-line therapy, 61 patients received second-line therapy, and 32 patients received third-line therapy or above. There were 30 patients who received pembrolizumab, 13 patients who received nivolumab, 2 patients who received atezolizumab, 17 patients who received durvalumab, 114 patients who received sintilimab, 66 patients who received camrelizumab, 28 patients who received toripalimab, 60 patients who received tislelizumab and 11 patients who received other ICIs.

### Clinical characteristics of patients with different nutritional risks

According to the different nutritional risks of NRS2002 before initiating the first immunotherapy treatment, all patients were categorized into two groups: one group without nutritional risk (NRS2002 0–2) and another group with nutritional risk (NRS2002 ≥ 3) as shown in Table 1. The median age of the group without nutritional risk was 64 years, and that of the group with nutritional risk was 70 years. Patients over 65 years old, with high NPS score or high LCR score, and esophageal cancer had a greater rate of malnutrition according to the NRS2002 score, and they also had a greater rate of receiving nutritional support treatment (Table 1).

**Table 1.** Clinical characteristics of 341 patients with different nutritional risks.

Clinical characteristics	NRS2002 ≥ 3 n(%)	NRS2002 0–2 n(%)	P-value
Age (years)			.001
≤65	23(31.94)	148(55.02)	
>65	49(68.06)	121(44.98)	
Gender			.743
Male	55(76.39)	198(73.61)	
Female	17(23.61)	71(26.39)	
PS*			.078
0	8(11.11)	57(21.19)	
1–2	64(88.89)	212(78.81)	
NPS**			.003
1	3(4.17)	52(19.33)	
2	69(95.83)	217(80.67)	
SII***			.117
Low	59(81.94)	241(89.59)	
High	13(18.06)	28(10.41)	
LCR****			
Low	23(31.94)	111(41.26)	.006
High	40(55.56)	95(35.32)	
Unknown	9(12.5)	63(23.42)	
Primary tumor location			.045
Bladder	1(1.39)	16(5.95)	
Breast	2(2.78)	3(1.12)	
Cervix	2(2.78)	6(2.23)	
Colorectal	6(8.33)	21(7.81)	
Easophage	16(22.22)	20(7.43)	
Biliary system	3(4.17)	8(2.97)	
Gastric	7(9.72)	23(8.55)	
Head and neck	1(1.39)	10(3.72)	
Liver	4(5.56)	5(1.86)	
Lung	20(27.77)	104(38.66)	
Multiple primary tumors	6(8.33)	22(8.18)	
Other	4(5.56)	27(10.04)	
Unknown	0(0)	4(1.48)	
Line of thrapy			.537
1	49(68.06)	199(73.98)	
2	16(22.22)	45(16.73)	
≥3	7(9.72)	25(9.29)	
Nutritional Support			<.001
No	31(43.06)	218(81.04)	
Yes	41(56.94)	51(18.96)	
Comorbidity			.097
No	36(50.00)	166(61.71)	
Yes	36(50.00)	103(38.29)	

*PS: Performance Status.

**NPS: Naples Prognostic Score.

***SII: System Immune Inflammation Index.

****LCR: Lymphocyte and C-reactive protein ratio.

### Survival analysis of patients receiving ICI treatment

**Figure 1.**
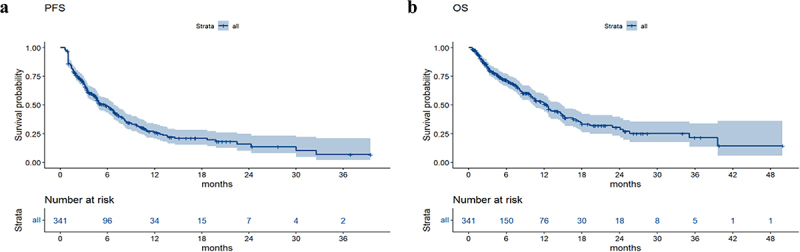
The Kaplan-Meier curves of PFS (a) and OS (b).

The median follow-up time was 4.9 months until June 30, 2022. In all patients, the median PFS was 5.8 months, and the median OS was 12.5 months (Figure 1).

**Figure 2.**
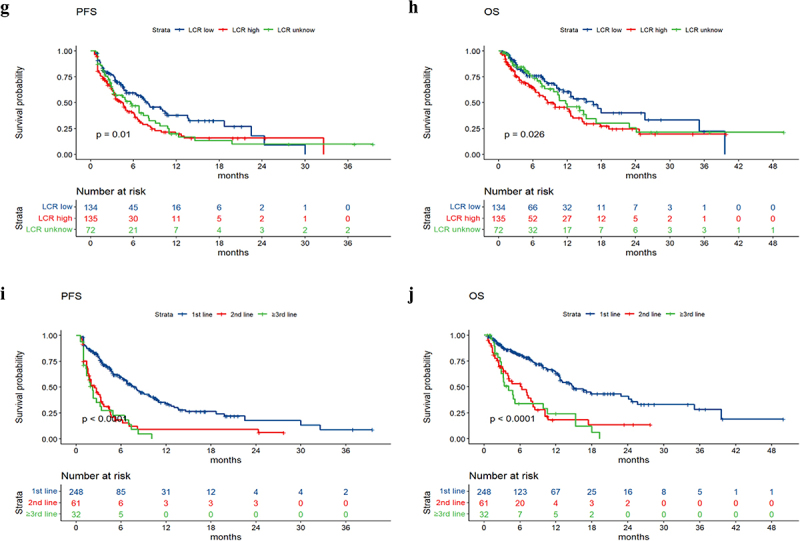
(Contiuned).

**Figure 3.**
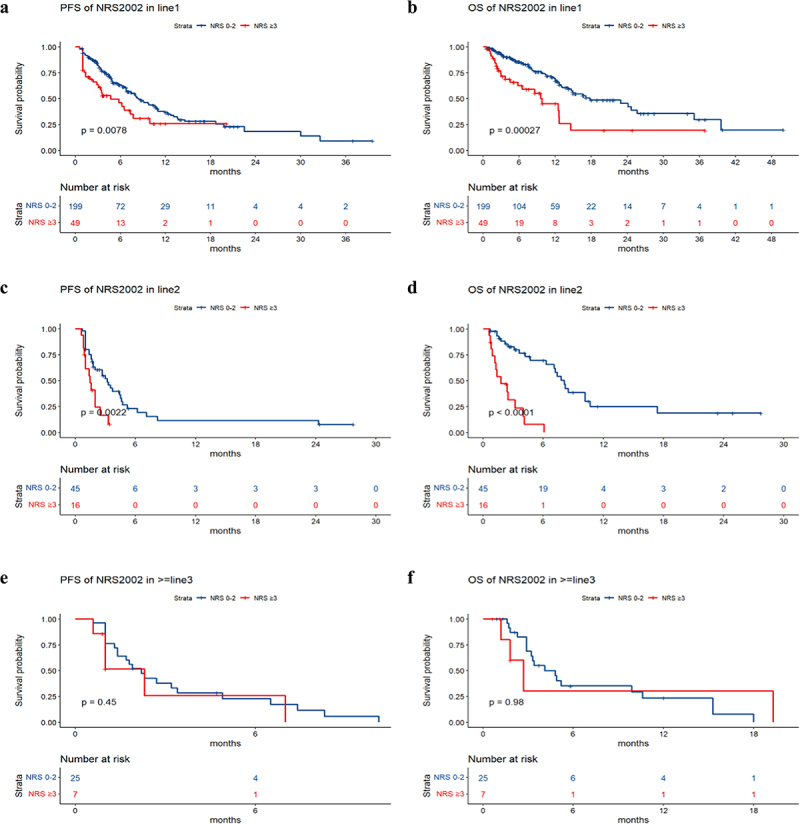
a-f the Kaplan-Meier curves of PFS and OS based on different treatment lines.

The median PFS values in the groups without nutritional risk and with nutritional risk were 6.6 months and 2.9 months, respectively. The median OS values in the groups without nutritional risk and with nutritional risk were 14.2 months and 4.5 months, respectively. Survival analysis revealed that patients with poorer PS (*p* value = .005), higher NRS2002 score (*p* value < .001), higher LCR score (*p* value = .01), and larger treatment lines (*p* value < .001) had poorer PFS, and patients with poorer PS (*p* value = .0075), higher NRS2002 score (*p* value < .001), higher NPS score (*p* value = .002), higher LCR score (*p* value = .026), and larger treatment lines (*p* value < .001) had poorer OS (Figure 2). The univariate and multivariate analyses for PFS and OS are shown in Tables 2 and 3. The PS, NRS2002 score, NPS, LCR, line of therapy, and nutritional support were significantly related to PFS or OS according to univariate analysis. According to the multivariate model, the PS, NRS2002 score and line of therapy were independent prognostic factors for PFS. The NRS2002 score, NPS, line of therapy and nutritional support were independent prognostic factors for OS. Stratified analysis of different lines revealed that NRS2002 score ≥ 3 was significantly related to worse OS and PFS relative to NRS2002 0–2 among patients receiving first-line treatment and second-line treatment, respectively (p < .01) (Figure 3). Therefore, the NRS2002 score was confirmed to be an independent prognostic factor for both PFS and OS.
**Figure 2.**
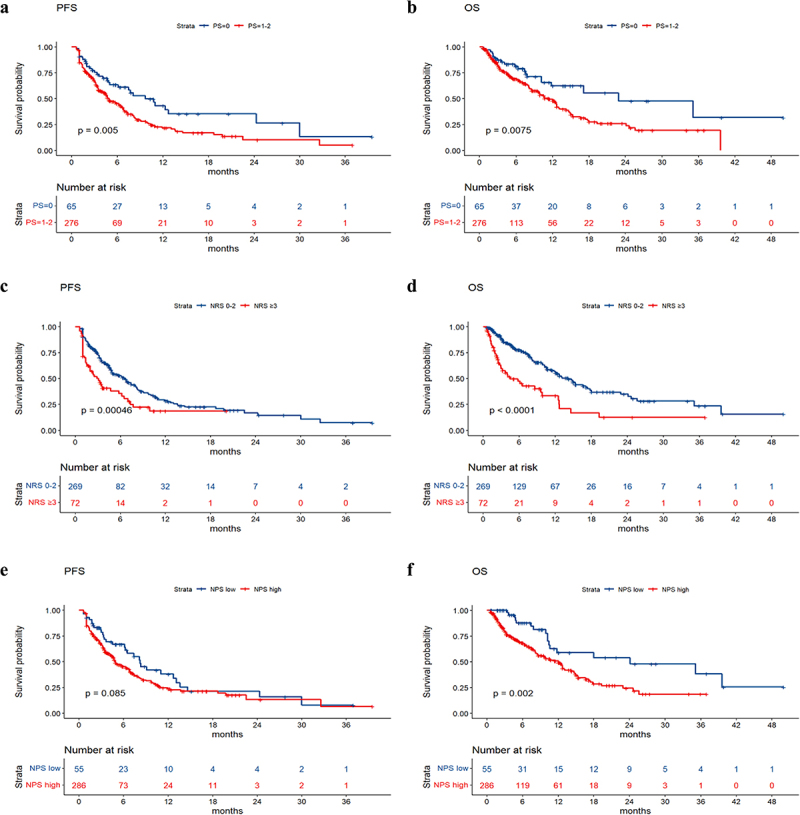
a-j the Kaplan-Meier curves of PFS and OS based on significant clinical factors.

**Table 2.** Univariate and multivariate analysis for PFS.

Variable	median PFS(m)(95%CI)	Univariate		Multivariate	
		HR(95%CI)	P-value	HR(95%CI)	P-value
Age (years)					
≤65	6.2(4.5–7.4)	1		–	
>65	5.6(4.5–7.7)	1.07(0.81–1.41)	.653	–	–
Gender					
Male	5.6(4.7–7.4)	1		–	
Female	5.8(3.4–8.3)	1.03(0.75–1.42)	.836	–	–
PS					
0	9.7(6.1-NA)	1		1	
1–2	4.9(4.2–6.5)	1.72(1.17–2.52)	.006	1.38(1.03–1.83)	.028
NRS 2002					
0–2	6.6(4.9–8.1)	1		1	
≥3	2.9(2.0–6.2)	1.82(1.30–2.54)	<.001	1.46(1.08–1.95)	.013
NPS					
1	8.2(6.2–13.6)	1		1	
2	4.9(4.4–6.8)	1.43 (1.07–1.91)	.016	1.32(0.97–1.80)	.080
SII					
Low	6.1(4.7–8.0)	1		–	
High	5.6(3.9–7.1)	0.97(0.78–1.20)	.770	–	–
LCR					
Low	8.0(6.3–13.6)	1		1	
High	4.4(3.3–6.1)	1.27(1.00–1.62)	.048	1.12(0.87–1.44)	.384
Unknown	5.6(3.2–8.2)	0.99(0.74–1.33)	.956	1.08(0.80–1.45)	.627
Line of thrapy					
1	7.7(6.6–9.9)	1		1	
2	2.5(1.7–3.5)	2.80(1.98–3.96)	<.001	1.91(1.43–2.55)	<.001
≥3	2.2(1.4–4.9)	3.52(2.30–5.38)	<.001	2.10(1.42–3.10)	<.001
Nutritional support					
No	6.8(5.8–8.3)	1		1	
Yes	2.5(1.9–4.1)	1.60(1.26–2.04)	<.001	1.22(0.92–1.61)	.172
Comorbidity					
No	5.8(4.6–7.4)	1		-	
Yes	5.0(3.7–8.2)	1.00(0.80–1.24)	.965	-	-

**Table 3.** Univariate and multivariate analysis for OS.

Variable	median OS(m)(95%CI)	Univariate		Multivariate	
		HR(95%CI)	P-value	HR(95%CI)	P-value
Age (years)					
≤65	12.0(10.2–16.6)	1		–	
>65	12.7(8.6–17.1)	1.02(0.74–1.43)	.892	–	–
Gender					
Male	12.4(9.9–14.6)	1		–	
Female	15.0(9.7-NA)	0.89(0.61–1.30)	.539	–	–
PS					
0	22.9(11.8-NA)	1		1	
1–2	11.4(9.6–13)	1.87(1.17–2.98)	.009	1.45(0.89–2.36)	.139
NRS 2002					
0–2	14.2(11.8–17.8)	1		1	
≥3	4.5(3.0–12.5)	2.49 (1.73–3.59)	<.001	1.61(1.05–2.47)	.028
NPS					
1	24.1(10.6-NA)	1		1	
2	12.0(8.6–14.2)	2.25 (1.33–3.82)	.003	1.84(1.05–3.23)	.033
SII					
Low	12.7(10.7–17.8)	1		–	
High	10.6(8.5–14.7)	1.23 (0.88–1.71)	.231	–	–
LCR					
Low	16.6(12.7-NA)	1		1	
High	9.6(7.1–12.7)	1.67(1.14–2.43)	.008	1.30(0.88–1.93)	.193
Unknown	11.8(9.7–22.9)	1.24(0.78–1.95)	.365	1.40(0.88–2.22)	.156
Line of thrapy					
1	14.9(12.7–24.6)	1		1	
2	6.3(4.0–8.2)	3.04(2.05–4.51)	<.001	3.40(2.27–5.09)	<.001
≥3	4.1(2.9–10.6)	3.38(2.12–5.39)	<.001	3.07(1.89–4.99)	<.001
Nutritional support					
No	14.9(12.7–24.1)	1		1	
Yes	4.1(3.1–8.6)	3.01 (2.15–4.21)	<.001	1.81(1.21–2.70)	.004
Comorbidity					
No	12.6(10.6–18.0)	1		–	
Yes	12.0(8.2–14.9)	1.25(0.90–1.74)	.185	–	–

### Objective tumor response to ICI treatment

Among the 341 patients, CR, PR, and SD were achieved in 3 (0.88%), 56 (16.4%) and 84 (24.6%) patients, respectively; one hundred ten (110) patients (32.3%) had progressive disease (PD), and the ORR was 17.3%. Among patients at nutritional risk, 0 (0%), 6 (8.33%), 14 (19.44%) and 30 (41.67%) achieved CR, PR, SD and PD, respectively. In contrast, the CR, PR, SD and PD for patients without nutritional risk were 3 (1.12%), 50 (18.59%), 70 (26.02%), and 80 (29.74%), respectively. There was no statistically significant difference in the above efficacy evaluation between the two groups (Table 4). However, the objective response rate (ORR) in the group at nutritional risk was greater than that in the group without nutritional risk (8.33% and 19.71%, respectively, *p* value = .037).

**Table 4.** Objective response of ICI treatment among different nutritional risk groups.

Objective response	Total(n)	NRS2002 ≥ 3 n(%)	NRS2002 0–2 n(%)	p-Value
CR	3	0(0)	3(1.12)	.104
PR	56	6(8.33)	50(18.59)	
NonCR/nonPD	88	22(30.56)	66(24.54)	
SD	84	14(19.44)	70(26.02)	
PD	110	30(41.67)	80(29.73)	
ORR(CR+PR)	59	6(8.33)	53(19.71)	.037

## Discussion

The discovery of the role and value of immune checkpoints in malignant tumors and the clinical research results showing substantial antitumour effects of the immune checkpoint inhibitors pembrolizumab and nivolumab in various solid tumors have been pivotal. Treatment with these and other ICIs has greatly improved the survival and prognosis of patients with advanced tumors, including those resulting from lung cancer, head and neck tumors, gastric cancer, and malignant melanoma.^12–15^ ICIs developed in China have reached their peak of research and development. To date, several such drugs have entered clinical use after their efficacy was confirmed in clinical trials. Antitumour therapy has truly entered the era of immunotherapy.

However, in general, patients in which immunotherapy is effective account for less than 50% of tumor patients,^15^ and more than half of patients have no response to immune checkpoint inhibitor agents. Predictive indicators of the efficacy of ICIs are still being explored. To date, it is unknown which clinical features of patients may indicate the potential for better or worse immunotherapeutic efficacy, and the exploration of biomarkers is ongoing. It is believed that the population with high PD-L1 expression, high tumor mutational burden (TMB) and high mismatch repair deficiency (dMMR) may be the dominant population that can benefit from immunotherapy. However, in clinical practice, even in patients with high PD-L1 expression, high TMB, or even dMMR, immunotherapy may not provide the expected significant benefits. Therefore, the predictive indicators of the efficacy of immunotherapy are not as clear as those of targeted therapy, and they still represent a mystery that has not been completely resolved. There is a long way to go to prove their value.

To observe the efficacy and related factors of immunotherapy in the real world, we retrospectively analyzed the clinical factors and efficacy of ICI therapy in 341 patients with solid tumors. The ORR in the group with NRS2002 score ≥ 3 was lower than that in the group with NRS2002 score between 0 and 2. Among the patients who received ICIs, those with NRS2002 score ≥ 3 had shorter survival times than those NRS2002 score between 0 and 2. According to multivariate analysis, the PS, NRS2002 score, NPS, line of therapy and nutritional support were independent prognostic factors for PFS or OS after ICI therapy.

A correlation between poor PS and poor survival after ICI therapy has been observed in many studies, including studies on lung cancer, urothelial carcinoma, and recurrent/metastatic head and neck cancer,^16–18^ and our cohort has provided verification. However, the reason that the physical status score affects the efficacy of immunotherapy is not clear. The explanation is most likely related to a weak physical performance status, resulting in a weak immune activation response. The influence of the nutritional status of tumor patients on survival has been confirmed. With regard to the relationship between the nutritional status of tumor patients and the efficacy of ICI therapy, a few researchers have found that the prognostic nutritional index (PNI) may be related to the efficacy of immunotherapy in gastric cancer,^19^ nasopharyngeal carcinoma,^20^ and lung cancer.^21,22^ A retrospective study showed that patients with non-small cell lung cancer and a low body mass index (BMI) who underwent ICI treatment had a lower median survival than those with a normal BMI or a higher BMI.^23^ Other researchers used the control nutritional status (CONUT) obtained from the serum ALB level, the total cholesterol level and the total lymphocyte count to evaluate the nutritional status of NSLSC patients and found that the CONUT score was an independent diagnostic factor in NSLSC patients treated with pembrolizumab.^24^ Unlike the above study, we used the NRS2002 score for nutritional risk assessment. The NRS2002 is a nutritional risk screening tool recommended by the European Society for Parenteral and Enteral Nutrition (ESPEN) for hospitalized patients, and it includes three factors: disease status, nutritional status (such as BMI, weight loss in 1–3 months, and dietary status within a week), and age.^25^ A total score ≥ 3 indicates nutritional risk. By asking tumor patients about their ages, weight changes, diets and BMI, the NRS2002 score can be quickly obtained, and this process is relatively simple and convenient. The correlation between nutritional status evaluated by the NRS2002 and tumor prognosis has been confirmed by multiple studies.^26–29^ The majority of studies have focused on postoperative or radiation therapy, while for immunotherapy, a relatively novel treatment method, the relationship between NRS2002 and the efficacy and prognosis of ICIs is still unclear. Chen Ning et al. explored the nutritional status of esophageal cancer patients to predict the efficacy and prognosis of immunotherapy. The results showed that pretreatment hemoglobin and body mass index were correlated with prognosis, but there was no significant difference in the NRS2002 score.^30^ Further research is necessary. The NPS was first proposed by Galizia et al. in 2017 based on nutritional and inflammatory status. The preoperative NPS is a reliable prognostic indicator for multiple tumors.^31,32^ Not surprisingly, patients who received more lines of treatment had lower PFS and OS. Moreover, through stratified analysis, there was significant difference in the survival of patients with different NRS2002 who received first-line treatment and those who received second-line treatment, respectively. Perhaps due to the small number of third-line treatment cases included, statistical significance could not be displayed. Survival also differed according to nutritional support treatment. We believe that this was due to poor nutritional status. Although nutritional support treatment was given, the prognosis of patients who received nutritional support was still worse than that of patients with good nutritional status.

Why does poor nutritional status affect the survival of patients receiving immunotherapy? With malnutrition, the nutrient absorption rates and glycolysis rates of tumor cells do not decrease, and their protein, lipid and nucleotide anabolism is still fast, markedly reducing availability of the nutrients required by immune cells; additionally, their proliferation is inhibited, ultimately weakening their killing function.^33^ In cases of severe malnutrition, the patient’s food intake is low, their intestinal function is weakened, and intestinal flora disorders may occur. A decrease in the gut microbiota may be a negative factor in the efficacy of ICIs.^34,35^ Severe malnutrition may be accompanied by metabolic stress, increasing the level of endogenous glucocorticoids and further inhibiting the efficacy of immunotherapy.^36^ Severe malnutrition may promote the release of inflammatory factors, such as TNF, IL-6, and IL-1.^37^ Inflammatory factor-mediated chronic inflammation may promote an immunosuppressive environment characterized by the activation of the immune checkpoint pathway in effector T cells.^38^ Of course, part of the reason may be that patients with poor nutritional status are often those receiving later lines of therapy; thus, the efficacy may be worse.

There are several limitations in our study. First, this was a retrospective study with a small sample size from a single medical center. We need to design a more rigorous prospective study. Second, our real-world study involved various tumor types, covering multiple lines of treatment. In the future, it may be necessary to determine a tumor type for research and determine the appropriate line of therapy. In addition, in clinical practice, with increasing emphasis on the nutrition of tumor patients, it is necessary to dynamically monitor the changes in the NRS2002 score and observe whether earlier and more active nutritional support to improve nutritional status can enhance the efficacy of immunotherapy.

## Conclusion

For patients at nutritional risk whose NRS2002 score is greater than or equal to 3, the benefit of ICI treatment may be inferior to that of patients whose NRS2002 score is 0–2. The results of this study suggest that we should pay more attention to the nutritional status of tumor patients at all times, conduct nutritional risk screenings and assessments on patients in a normalized manner, and provide nutritional interventions for patients at nutritional risk as early as possible to improve their nutritional status, thus enhancing immunotherapy efficacy, survival time and prognosis.

## Data Availability

The data in this study can be obtained from the corresponding author’s e-mail address (11218276@zju.edu.cn).
